# Early-life RSV infection modulates innate immune events, preferentially enhancing allergen-induced type 2 lung inflammation in females

**DOI:** 10.1371/journal.ppat.1013340

**Published:** 2025-07-21

**Authors:** Lydia Labrie, Rojine C. McVea, Rami Karkout, Haya Aldossary, Véronique Gaudreault, Brian J. Ward, Elizabeth D. Fixman

**Affiliations:** 1 Meakins-Christie Laboratories, Research Institute of the McGill University Health Centre, Montréal, Québec, Canada; 2 Research Institute of the McGill University Health Centre, Montréal, Québec, Canada; University of Pittsburgh School of Medicine, UNITED STATES OF AMERICA

## Abstract

Respiratory syncytial virus (RSV) causes millions of hospitalizations and thousands of deaths per year globally. Early-life RSV infection is also associated with the subsequent development of wheezing and asthma, which exhibits sex-related disparities in incidence, epidemiology, and morbidity. The mechanisms that underlie these sex-specific effects are not clear. We have developed a combined infection-allergy model in which 10-day old mice are infected with RSV and subsequently exposed to a common allergen, house dust mite (HDM). We show that early-life exposure to RSV enhanced allergic lung inflammation upon HDM exposure 10 days after viral infection. Early-life RSV infection increased levels of the innate cytokine, IL-33, in the lung 6h following HDM exposure. Accumulation of CD11c^med^ eosinophils and group 2 innate lymphoid cells was more prominent in the lungs of female mice exposed to both RSV and HDM. Moreover, the numbers of IL-13^+^ T cells (both CD4^+^ and CD8^+^) in the lung were significantly increased in mice exposed to both RSV infection and HDM, although the expression of ST2 (the cognate receptor for IL-33) was not linked to T cell cytokine production. Inflammatory responses were maintained when the interval between RSV infection and HDM exposure was extended to one month. Thus, our results show that early exposure to RSV increased numbers of innate cells as well as T cells in response to a common allergen, whether delivered within days or after several weeks of viral infection and that most responses were enhanced in female mice. Our work highlights sex-specific impact of early-life viral infection on the developing lung, and suggests possible mechanisms to explain the subsequent predisposition to enhanced allergic responses long after viral clearance.

## Author summary

Respiratory syncytial virus (RSV) is one of the main causes of lower respiratory tract infection, affecting mainly young children and the elderly. RSV infection in infants is associated with an elevated risk of developing wheezing and asthma, with important sex-related disparities in incidence and morbidity, through mechanisms that remain poorly understood. We have developed a novel combined infection-allergen exposure model in which 10-day old mice are infected with RSV and subsequently, 10 days later, exposed twice over two days to a common allergen, house dust mite (HDM). While this acute exposure to HDM alone had little effect on lung inflammation, in mice previously infected with RSV, type 2 allergic lung inflammation was enhanced. Moreover, inflammatory responses in the lung were greater in females compared to males. When the interval between RSV infection and HDM exposure was extended to one month, inflammation was still enhanced in females, now accompanied by airway hyperresponsiveness. Thus, our work highlights the sex-specific impact of early-life viral infection on the developing lung and suggests possible mechanisms to explain the subsequent predisposition to enhanced allergic responses long after viral clearance.

## Introduction

Respiratory syncytial virus (RSV) is a leading cause of acute lower respiratory tract infections, with a recorded 33 million episodes of lower respiratory infections in children under 5 years of age; RSV causes approximately 118,000 deaths in children annually worldwide with 99% occurring in low- and middle-income countries [1–3]. During the first year of life, ~ 70% of infants are infected with RSV and nearly all children are infected at least once by two years of age [1,4]. Epidemiological data associate early-life RSV infection with a heightened risk of asthma development [5–8], with almost half of the infants hospitalized with RSV bronchiolitis developing asthma later in life [8]. Roughly 5 million children in the United States alone are affected by asthma of whom 1.3 million are under the age of five [9]. However, the mechanisms underlying the association between early RSV infection and asthma are unclear.

Several murine models have been developed to explore the ability of RSV infection to enhance responses to allergens [10–12]. Keegan et al. infected adult mice with RSV prior to challenging them multiple times with an extract of house-dust mite (HDM) and demonstrated that RSV infection increased HDM-induced eosinophil influx into the airways as well as mucus production in the lung [10]. Numerous groups have shown that age of initial exposure to RSV plays an important role in influencing immune responses upon RSV reinfection [13,14]. Age is also an important factor in RSV enhancement of responses in asthma models. When You et al. infected neonatal mice and subsequently sensitized and challenged these animals with ovalbumin as adults, they observed exacerbated airway hyperactivity (AHR) and inflammation with subepithelial fibrosis [12]. Similarly, Malinczak et al. showed that neonatal RSV infection followed by sensitization and challenge with cockroach allergen extract (CRE) in adults increased mucus production and AHR, predominately in adult male mice [11]. Severe RSV infection in children is also associated with enhanced T-helper 2 (Th2) polarization in the lung with exacerbated IL-4 and IL-13 levels [15–19]. While murine models appear to recapitulate the effect of RSV infection on adaptive immune responses following allergen sensitization and challenge in adult mice, how RSV alters innate immune events prior to the onset of allergen-induced Th2 adaptive immunity is not known.

It is well established that sex-related differences exist in asthma epidemiology and morbidity, although the mechanisms that underlie these differences are poorly understood [20]. The prevalence of asthma is greater in young males but switches around the time of puberty, when asthma prevalence becomes greater in females [20]. In agreement with these clinical observations, data from animal models suggest that inflammatory responses in asthma are enhanced in (adult) female mice [21–23]. Notably, group 2 innate lymphoid cells (ILC2s), which are implicated in both asthma and severe RSV infections, are negatively controlled by androgens [23–26].

Genome-wide association studies (GWAS) demonstrate an association between the innate alarmin cytokine, IL-33, or its cognate receptor subunit, ST2, and risk for both asthma and severe RSV disease [27,28]. Blocking IL-33, which is released upon RSV infection in neonatal mice, reduces both ILC2 expansion in neonates and disease severity upon RSV reinfection of adults [24]. Two therapies that reduce severe RSV infection in infants have recently been approved: maternal RSV vaccination and a long-acting RSV monoclonal antibody [29]. While greeted with great enthusiasm, there is little evidence that these interventions prevent RSV infection per se, and thus it remains unclear if they will reduce long-term outcomes (i.e., wheezing, asthma) linked to early-life RSV exposure. Thus, novel preventative and therapeutic strategies that reduce the health burden of RSV, and subsequent development of asthma, have the potential to impact hundreds of thousands globally.

In this work, we present a new RSV infection model we developed in order to define the early inflammatory events induced upon acute 2-day allergen exposure in mice previously infected with RSV. Our goal was to examine type 2 inflammation prior to the onset of allergen-induced Th2 adaptive immunity. We hypothesized that neonatal RSV infection would enhance type 2 innate inflammatory responses induced within days of an acute exposure to HDM. Our data show that early-life exposure to RSV dramatically enhanced HDM-induced lung eosinophil influx and activation, ILC2 expansion, as well as levels of type 2 cytokines (IL-13, IL-5) that orchestrate pathology in the asthmatic lung. Our data also demonstrate that a subset of these responses was more prominent in female mice. Early-life RSV infection also dramatically enhanced acute HDM-induced type 2 inflammatory responses, including AHR, long after viral clearance (4 weeks after initial infection). Together, these results highlight how early-life RSV infection may modulate innate immunity in the lung to promote type 2 inflammation and the subsequent development of asthma.

## Materials and methods

### Ethics statement

Animal studies were approved by the McGill University Animal Care Committee under protocol number MUHC-8082 and performed in accordance to the guidelines of the Canadian Council on Animal Care.

### Mice

Wild-type BALB/c mice (originally from Charles River Laboratories, St-Constant, QC) were bred in-house under pathogen-free conditions. Breeding trios were used (2 females, 1 male). Once pregnant, dams were housed one per cage and transferred to CL2 facilities. Male and female pups used for experiments were housed with their mother until day 21 of life, when they were weaned and males and females separated into different cages. All cages were supplemented with water and irradiated food at all times.

### RSV purification

RSV A2 (ATCC #VR-1540, Manassas, VA) was used to infect Hep-2 cells (ATCC #CCL-23, Manassas, VA). The virus was added to RPMI-1640 medium containing 2.5% heat inactivated fetal bovine serum (FBS) (Wisent, St-Bruno, QC), mixed gently, then added to 70–80% confluent Hep-2 cells in a 24-well plate. While placed on a rocker at 4˚C, the virus was adsorbed to the monolayer for 90 minutes, after which the monolayer was washed with RPMI + 2.5% FBS and cultured at 37°C in fresh RPMI + 2.5% FBS for 3 days. Once the cell monolayer exhibited cytopathic effect (CPE) (40–50%) under light microscopy, the monolayer was disrupted by scraping. Supernatant was collected after two washes (once with H_2_O, once with supernatant from the first wash, spun at 2000rpm, 5 minutes, 4˚C) and then transferred to a T175 flask containing 80–90% confluent Hep2-cells. Virus was adsorbed for 90 minutes at 4˚C on a rocker. The monolayer was washed with RPMI + 2.5% FBS and cultured at 37°C in fresh RPMI + 2.5% FBS for 3 days. When CPE reached 70–80%, the cell monolayer was disrupted by scraping (as above) and the supernatant clarified from cellular debris by centrifugation at 4°C for 10 minutes at 2095 x g [30]. The virus was further purified in a sucrose gradient with centrifugation at 116 000 x g for 2 hours at 4°C [31]. Purified virus was aliquoted and stored at -80°C. Viral titers were determined by TCID_50_ as previously described [31].

### RSV infection and quantification and HDM exposure

Wild-type BALB/c mice (four- or ten-days of age) were briefly anesthetised under isoflurane and treated intranasally (i.n.) with 10µL of RSV A2 (10^6^ TCID_50_/g body weight) or mock solution (obtained through the same process as “*RSV purification”*, except in the initial step virus-free RPMI medium + 2.5% FBS was added to Hep-2 cells). Mice were sacrificed at various time points to quantify virus by RT-qPCR as previously [32]. For mice younger than 20 days, the entire right lung was collected; for mice aged 20 days, only the right inferior lobe was harvested. Lung tissues were snap-frozen in liquid nitrogen and stored at −80 °C until processing. Total RNA was extracted from frozen lung samples using TRIzol reagent (Ambion, Carlsbad, CA) according to the manufacturer’s protocol. Complementary DNA (cDNA) was synthesized from 1 µg of total RNA using the iScript cDNA Synthesis Kit (Bio-Rad) following the manufacturer’s instructions. qPCR amplification of the RSV *NS1* gene was performed using SYBR Green Master Mix (Bio-Rad) with the following primers: *NS1* forward, 5′-CACAACAATGCCAGTGCTACAA-3′; *NS1* reverse, 5′-TTAGACCATTAGGTTGAGAGCAATGT-3′. Levels of RNA were normalized to β-actin using the following primers: β-actin forward, 5′-AGCCATGTACGTAGCCATCC-3′; β-actin reverse, 5′-CTCTCAGCTGTGGTGGTGAA-3′. Thermal cycling conditions included an initial denaturation at 95 °C for 30 seconds, followed by 40 cycles of 95 °C for 15 seconds and 60 °C for 30 seconds.

Levels of NS1 were calculated with the ΔΔCt method, normalized to beta-actin, and presented relative to levels of mock-infected control mice. Samples with undetectable *NS1* expression were assigned a Ct value of 40 for analysis. For HDM exposure, ten or thirty days post RSV infection, mice were briefly anaesthetized again prior to delivery of 50µg of low-endotoxin HDM extract (Stallergenes Greer Ltd, London, UK) in a 20µL solution or an equal volume of phosphate buffered saline (PBS) i.n. (equally between both nares) on each of two consecutive days.

### Lung digestion and preparation of cells for flow cytometry

Lungs were collected and cut into small pieces using sterile scissors and digested enzymatically for 30 minutes at 37˚C, 5% CO_2_ with a cocktail of DNase I (200µg/ml; Sigma-Aldrich, St. Louis, MO), Liberase (100µg/ml; Roche, Indianapolis, IN), hyaluronidase 1a (1mg/ml; Life Technologies, Carlsbad, CA), and collagenase XI (250µg/ml; Life Technologies, Carlsbad, CA) in RPMI-1640 as described previously [33]. Cells were then washed with RPMI-1640 media containing 1% Penicillin/Streptomycin and 5% FBS. Sterile, filtered ammonium-chloride-potassium (ACK) buffer was used to lyse red blood cells. Finally, filtration through a 0.7 μM strainer was performed and the remaining viable cells were recovered.

Trypan blue exclusion was used to enumerate viable cells and samples were diluted to obtain 1 × 10^6^ cells for eosinophil/macrophage staining, 2 × 10^6^ cells for ILC2 staining, and 1.5x10^6^ cells for T cell staining. For T cell staining, cells were stimulated for 4 hrs, at 37˚C, 5% CO_2_ with phorbol 12-myristate 13-acetate (PMA) (0.5µg/ml, Sigma-Aldrich, St. Louis, MO) and ionomycin (1 µg/ml, Sigma-Aldrich, St. Louis, MO) in the presence of GolgiStop™ (0.133µl/ml; BD Biosciences, Franklin Lakes, NJ). ILC2s were incubated with only GolgiStop™ (0.133µl/mL). Cells were washed three times with PBS or FACS buffer between staining steps. All cells were incubated in the dark for 20 min with eFluor780 viability dye (eBioscience, San Diego, CA), then incubated at 4°C for 10 min with anti-CD32/16 to block Fc receptors (Mouse BD FC block, BD Biosciences, Franklin Lakes, NJ). Macrophages and eosinophils were stained together with the following antibody cocktail: BUV395-CD45.2, Alexa Fluor 700-Ly6G, APC-F4/80, Alexa Fluor 488-CD11c, PeCy7-CD11b, PE-Siglec F. Interstitial macrophages (IM) were defined as CD45.2^+^, Ly6G^+^, CD11c^+^, F4/80^+^, CD11b^+^ and alveolar macrophages (AM) were defined as CD45.2^+^, Ly6G^+^, CD11c^+^, F4/80^+^, CD11b^-^ (S1A Fig). Eosinophils were defined as CD45.2^+^, Ly6G^+^, SiglecF^+^, CD11c^med^ or CD11c^-^ (S1A Fig), all of which expressed CD11b. ILC2s were stained using EF-450-Thy1.2, PECy7-CD127, PerCP-eF710-ST2, KLRG1-BV605, CD45.2-BUV395, BV510-MHCII and a combination of PE-conjugated antibodies to CD3e, CD11c, CD11b, CD49b, CD45R, TCRyD, Ly6G, and FCeRa1 (S2A Fig). T cells were stained using FITC-CD4, PerCP-Cy5.5-CD8, V500-CD3, BUV395-CD45.2 (S2B Fig). Cells were then fixed with intracellular (IC) fixation buffer (eBioscience, San Diego, CA) overnight. Afterward, ILC2s and T cells were permeabilized with BD Perm/Wash buffer (BD biosciences, Franklin Lakes, NJ). ILC2s were stained with AF488-IL-13 and APC-IL-5 while T cells were stained with APC-IL-4, PE-IL-13, BV421-IL-5. All cells were acquired using the BD LSRFortessa (Immunophenotyping Core Facility, RI-MUHC) flow cytometer. Analysis was completed with FlowJo V10 (FlowJo LLC, Ashland, OR). To define positive populations, fluorescence minus one (FMO) controls were used (see insets, S2A and S2B Fig). Additional information for the antibodies can be found in A-C_Tables in S1 File.

### Lung explant culture

For lung explant culture, 2 million viable cells were allocated to two conditions, saline or IL-33. In 1mL of RPMI-1640 media with 10% FBS, 5% penicillin/streptomycin, 1mM sodium pyruvate, 1mM non-essential amino acids, and 55µM 2-mercaptoethanol containing either saline or IL-33 (20ng/mL) cells were incubated at 37˚C and 5% CO_2_ for 48 hours, as previously shown [34,35]. Supernatants were then collected and ELISA was used to quantify IL-13 and IL-5.

### IL-13 and IL-5 ELISA

IL-13 and IL-5 were quantified using Ready-SET-Go! IL-13 and IL-5 ELISA kits from Thermo Fisher Scientific (Carlsbad, CA) following the instructions provided. In duplicate, samples from cells cultured in saline were used neat. Samples cultured in IL-33 from mice treated with RSV and HDM were diluted 1/100, while those from mice treated with HDM only, RSV only and PBS only were diluted 1/50 to ensure values fell within the detection limit of the assay, 3.5 to 500 pg/mL.

### IL-33 ELISA

A piece from the lower right lung lobe, frozen in liquid nitrogen and then stored at -80˚C, was placed in cold lysis buffer (20mM Tris-HCL, 0.14mM NaCl, 10% glycerol, 10% protein inhibitor [Sigma*Fast*, Oakville, On, Protease Inhibitor Cocktail Tablet diluted 1/10 in H_2_O to dissolve the tablet]). Tissue was homogenized using Fisher’s homogenizer 150 at max speed for 10 seconds, twice after which 55 µL of 10% Igepal (CA-630, Sigma-Aldrich, St. Louis, MO) was added to each sample. Samples were centrifuged 12000rpm for 15 minutes at 4˚C. Supernatants were collected and total protein quantified (Bradford reagent, Sigma-Aldrich, St. Louis, MO). IL-33 mouse ELISA kit from Thermo Fisher Scientific (Carlsbad, CA) was used to quantify IL-33 following the manufacturer’s instructions. In duplicate, samples from mice treated with RSV and HDM or HDM only were diluted 1/20, while those from mice treated with RSV only or PBS only were diluted 1/10 to ensure that values obtained fell within the detection limit of the assay, 25–3000 pg/mL.

### Lung function measurements

Lung function was assessed in adult mice using the flexiVent small animal ventilator (Scireq, Montréal, QC) as described previously [36,37]. Briefly, mice were anesthetized using xylazine and sodium pentobarbital and paralyzed with pancuronium bromide. Mice were attached to the flexiVent apparatus. Baseline respiratory system resistance, elastance, and Newtonian resistance as well as maximal respiratory system resistance, elastance, and Newtonian resistance to increasing doses of nebulized methacholine were recorded.

### Histology

Lungs were perfused with PBS then inflated through the trachea with 10% formalin. The entire lungs were isolated and placed in formalin. Lungs were embedded in paraffin and sections of 0.5μm were cut and mounted on glass slides, which were stained with haematoxylin and eosin (H&E). Slides were examined under a microscope (Zeiss Axio Imager M2, Wetzlar, Germany) and quantified by 2 individuals blinded to the groups, scoring inflammation on a scale from 1-3, based on the level of inflammation.

### Statistical analysis

Graphpad Prism 9 Software (San Diego, CA) was used for all analyses. Data were analyzed by one-way ANOVA or two-way ANOVA as described in the Figure Legends. Tukey’s *post hoc* test was used for multiple comparisons. A p ≤ 0.05 was considered significant. To remove outliers Grubb’s test with an alpha of 0.05 was used.

## Results

### Early-life INFECTION with RSV enhances lung eosinophil responses to HDM in female mice

**Fig 1:**
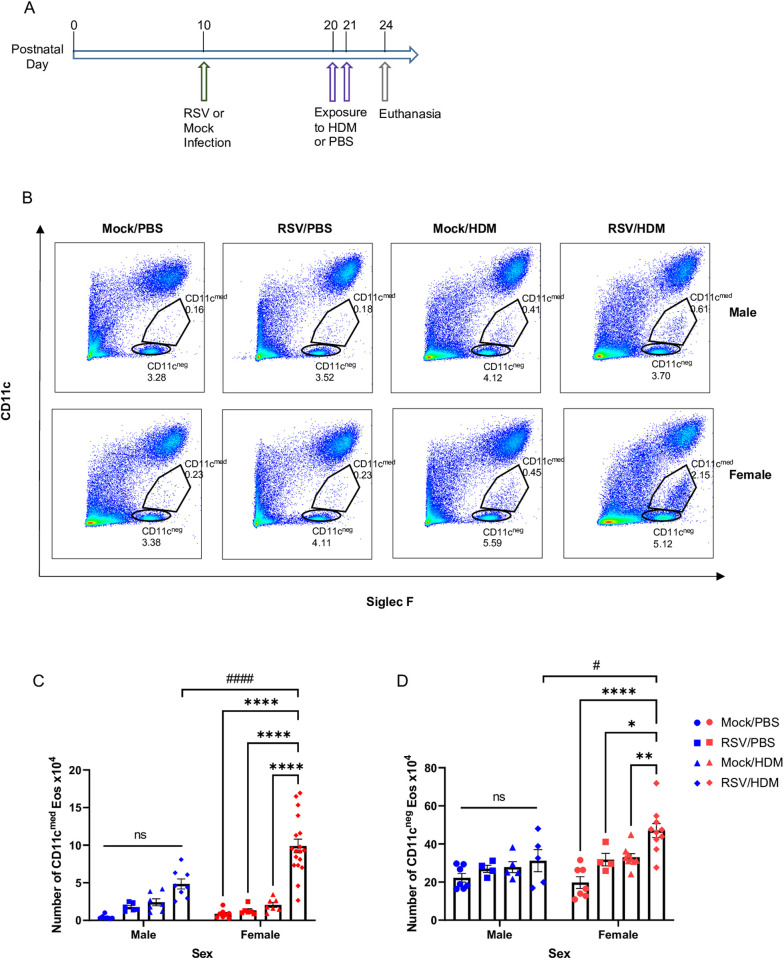
Early-life infection with RSV enhances lung eosinophil responses to HDM in females. **(A)** Mice were mock infected or exposed to RSV intranasally on PND10 and then treated with PBS or HDM (50ug per dose) intranasally on PND20 and PND21. Seventy-two hours later, lungs were harvested. **(B)** Representative flow cytometry panels highlighting CD11c^–^ and CD11c^med^ eosinophil populations for each treatment in each sex. **(C, D)** Absolute count of CD11c^med^ and CD11c^–^ eosinophils in the lung. Blue for male, red for female. Data are from the combination of four independent experiments (n = 4–18 per group). Outcomes are presented as mean ± SEM assessed by two-way ANOVA, Tukey’s post hoc test. ns = not significant, * p ≤ 0.05, **or ## p ≤ 0.01, **** or #### p ≤ 0.0001.

An acute RSV → HDM model was developed to investigate whether early-life RSV infection enhanced HDM-dependent type 2 innate inflammatory responses (Fig 1A). Male and female mice were infected with RSV at day 10 of life (PND10), a time when the neonatal mouse lung is skewed toward a type 2 inflammatory response in an IL-33-dependent manner [38]. Afterward, when RSV was no longer detectable (S3 Fig) on PND20 and 21, mice were exposed to HDM. Three days later, prior to the onset of Th2 adaptive immunity, lung inflammatory responses were assessed. While lung neutrophils were increased in all mice exposed to HDM, they were not enhanced in mice previously infected with RSV; no differences were noted in alveolar macrophages (S1B and S1C Fig). Although the number of interstitial macrophages was increased in RSV → HDM mice compared to Mock→HDM or RSV → PBS mice, sex differences in the number of these cells were not observed (S1D Fig). Lung eosinophil accumulation and upregulation of CD11c, as a marker of activation [34,39], were also examined by flow cytometry (Fig 1B) using a gating strategy described by Abdala-Valencia, who showed that CD11c-negative eosinophils increase in the lung where they upregulate CD11c and transit into the airway lumen [40]. Our data show that activated CD11c^med^ eosinophils were significantly increased in RSV → HDM female mice compared to RSV → PBS or Mock→HDM female mice (Fig 1C). The increase in CD11c^med^ activated eosinophils was also significantly higher in female mice compared to their male counterparts (Fig 1C). Eosinophils lacking CD11c expression were also significantly increased in female mice that received both RSV and HDM compared to all other groups (Fig 1D). Altogether, these data show that RSV infection followed by acute HDM exposure 10 days later enhanced eosinophil recruitment and activation selectively in female mice, while responses in other innate cells did not exhibit sex differences and were less pronounced.

### Early-life RSV infection enhances HDM-induced ILC2 responses, selectively in female mice

**Fig 2:**
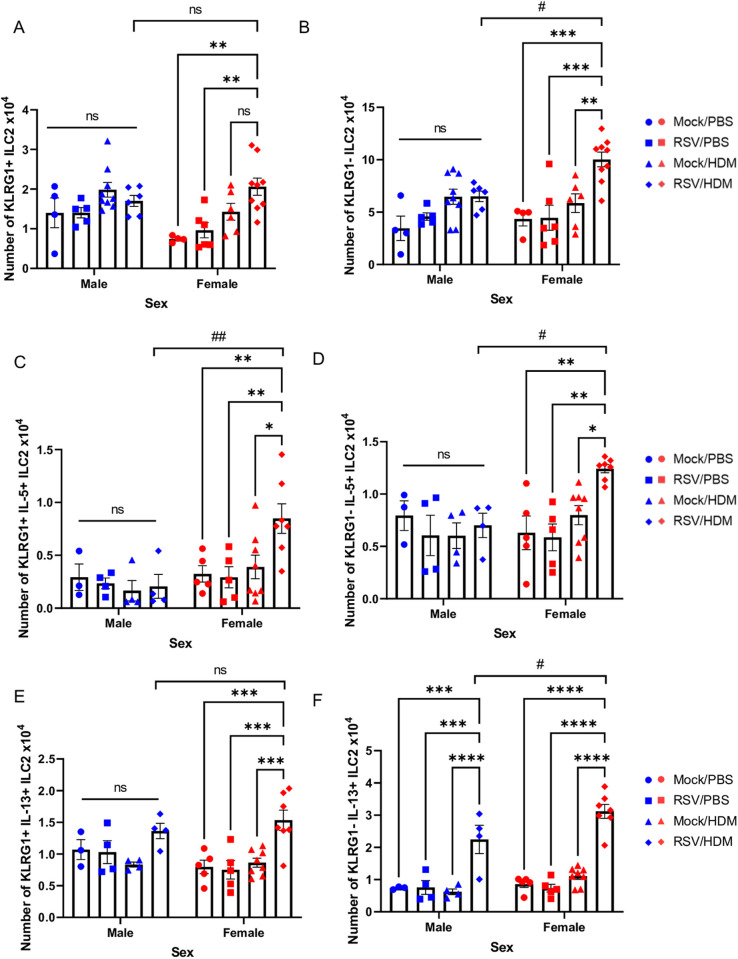
Early-life RSV infection enhances HDM-induced ILC2 responses, selectively in females. Mice were treated as in Fig 1A. Absolute count of **(A)** KLRG1^+^ ILC2s and **(B)** KLRG1^–^ ILC2s in males and females. Absolute count of KLRG1^+^ ILC2s expressing **(C)** IL-5 or **(E)** IL-13 in males and females. Absolute count of KLRG1^–^ ILC2s expressing **(D)** IL-5 or **(F)** IL-13 in males and females. Blue for male, red for female. Data are from the combination of two independent experiments (n = 4–7 per group). Outcomes are presented as mean ± SEM assessed by two-way ANOVA, Tukey’s post hoc test. ns = not significant, *or # p ≤ 0.05, **or ## p ≤ 0.01, *** p ≤ 0.001, **** or #### p ≤ 0.0001.

To better understand how RSV enhanced HDM-induced eosinophil responses selectively in female mice, we examined ILC2 responses. We focused on ILC2s because these cells produce large amounts of type 2 cytokines, IL-13 and IL-5, in response to innate cytokines, including IL-33, and exhibit enhanced responsiveness in adult female mice [21,23,41]. Importantly, RSV infection in neonatal mice (PND4) is associated with an increase in ILC2 numbers [24]. Our data demonstrate that a subset of ILC2s were selectively enhanced in female mice exposed to RSV → HDM (Fig 2). More specifically, the number of KLRG1^–^ ILC2s, but not KLRG1^+^ ILC2s, was significantly increased in RSV → HDM female mice compared to RSV → PBS or Mock→HDM female mice (Fig 2A and 2B). Both KLRG1^–^ and KLRG1^+^ ILC2s are functional; however, interactions between E-cadherin, which is expressed by epithelial cells, and KLRG1 on ILC2s, can dampen ILC2 responsiveness, at least in vitro [42,43]. These data suggest that prior RSV infection in young female mice has a lasting impact on ILC2s, particularly those lacking expression of KLRG1, which selectively expand upon subsequent exposure to HDM. Similar responses were not observed in male mice as ILC2 number (whether KLRG1^–^ or KLRG1^+^), did not significantly differ in mice exposed to RSV with or without later HDM (Fig 2A and 2B). The number of IL-5^+^ ILC2s (whether KLRG1^–^ or KLRG1^+^) was significantly increased only in RSV → HDM female mice (Fig 2C and 2D). While the number of IL-13^+^ KLRG1^+^ ILC2s was significantly increased in female, but not male, RSV → HDM mice, compared to RSV → PBS or Mock→HDM mice, there was no difference between males and females within this group (Fig 2E). In addition, although the number of IL-13^+^ KLRG1^–^ ILC2s was greater in both male and female RSV → HDM mice, the number of these cells was still significantly greater in females (Fig 2F). Similar to the increase in absolute number of cytokine-producing ILC2s, the percent of ILC2s within the parent population (whether KLRG1^+^ or KLRG1^–^) producing IL-5 or IL-13 was also increased (S4A-S4D Fig), providing evidence that the overall increase in cytokine-producing ILC2s, particularly in females, was due to a greater propensity of the ILC2s to produce IL-5 or IL-13. Thus, these female-specific enhanced ILC2 responses may be responsible, at least in part, for the greater recruitment and activation of eosinophils in female mice.

### Early-life RSV infection enhances HDM-induced release of IL-33

**Fig 3:**
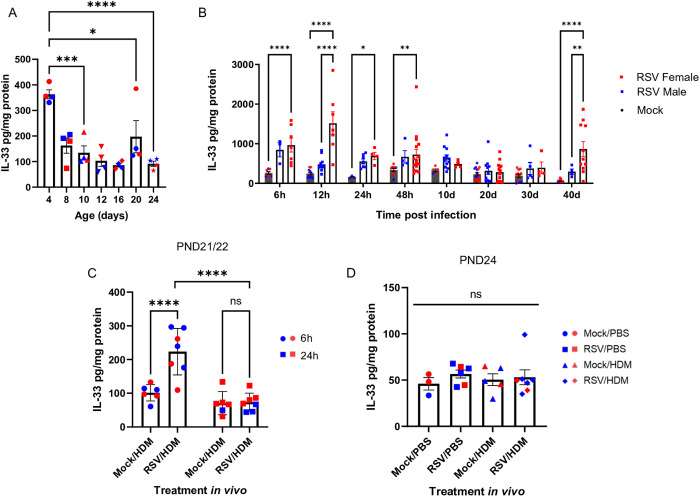
RSV infection enhances HDM-induced release of IL-33. **(A)** Levels of IL-33 in the lung of untreated mice at various ages, indicated on the x-axis. **(B)** Levels of IL-33 in the lung of PND10 mice treated with PBS or exposed to RSV and sacrificed from 6 hours to 40 days after RSV exposure. **(C)** Mice were treated as in Fig 1A, except they were sacrificed 6h or 24h after HDM to quantify levels of IL-33 in the lung. **(D)** Mice were treated as in Fig 1A and levels of IL-33 in the lung quantified 72h after HDM. Blue for male, red for female. Data in A, C, and D are from a single experiment for each panel with n = 4–7 per group. Data in B are compiled from 4 experiments with n = 3-16 per group. Outcomes are presented as mean ± SEM assessed by two-way ANOVA, Tukey’s post hoc test. ns = not significant, *p ≤ 0.05, **p ≤ 0.01, ***p ≤ 0.001, ****p ≤ 0.0001.

IL-33 is constitutively expressed in the nuclei of endothelial and epithelial cells [44]. Following cellular damage or necrotic cell death, full-length IL-33 is released [45]. Allergens with protease activity, including HDM, can both induce the rapid release of IL-33 as well as process IL-33 into shorter, more biologically active mature forms [46]. In order to better understand whether IL-33 release was associated with enhanced inflammatory responses to HDM in mice previously exposed to RSV, we quantified lung IL-33 levels in mice exposed to RSV and/or HDM at various time points. Consistent with data from Lambrecht and colleagues [38], in untreated mice, IL-33 levels in the lung were highest on PND4, after which they decreased by PND8 and remained constant until at least PND24 (Fig 3A). Saravia et al have shown that after RSV infection of PND4 neonates, IL-33 levels in the lung peak at 6 hours post-infection [24]. Similarly, we found that in PND10 female mice infected with RSV, IL-33 levels were greater at 6 hours and remained elevated up to at least 48h, before returning to baseline 10 days later at PND20, at the time of HDM delivery. Similar trends were also noted in RSV-infected males (Fig 3B). Interestingly, IL-33 levels were significantly increased 6h after the second HDM exposure, selectively in mice that had previously been infected with RSV, though the amount of IL-33 released did not differ between males and females (Fig 3C). There were no differences in IL-33 levels 72 hours following HDM delivery between any of the groups, providing evidence that IL-33 levels were not elevated concurrently with increased eosinophils and ILC2s (Fig 3D). We also quantified TSLP in these experiments, but it was not detectable at any of the above time points.

Given the data demonstrating an impact of prior RSV infection on HDM-induced responses on eosinophils and ILC2s, both of which express ST2 and have the ability to respond to IL-33, we hypothesized that ex vivo cytokine production from total lung cells in response to IL-33 would be enhanced in RSV → HDM mice. Thus, total lung cell populations were cultured ex vivo with either PBS or IL-33 and production of IL-13 and IL-5 was quantified. While levels of each cytokine were modest in cells cultured with saline, readily detectable amounts of both cytokines were produced upon culture with IL-33 regardless of their exposure history (i.e.,: PBS → PBS, RSV → PBS, Mock→HDM or RSV → HDM) (S5A and S5B Fig). Nevertheless, lung cells from RSV → HDM mice produced significantly greater amounts of both IL-13 and IL-5, compared to all other groups (S5A and S5B Fig). These data suggest that the profile of IL-33-responsive cells was increased in RSV → HDM mice, although cytokine production did not differ in cells harvested from males and females. Altogether, these data suggest that early-life RSV infection not only promoted acute release of IL-33 in the lung, but also shaped the lung environment so that, upon subsequent allergen exposure, larger amounts of IL-33 were released. Nevertheless, because the levels of IL-33 did not differ between males and females, these data suggest that female-specific enhanced responses are downstream of IL-33 and/or that other factors promote enhanced responses in female mice.

### Early-life RSV infection enhances Th2 and Tc2 cell responses to HDM

**Fig 4:**
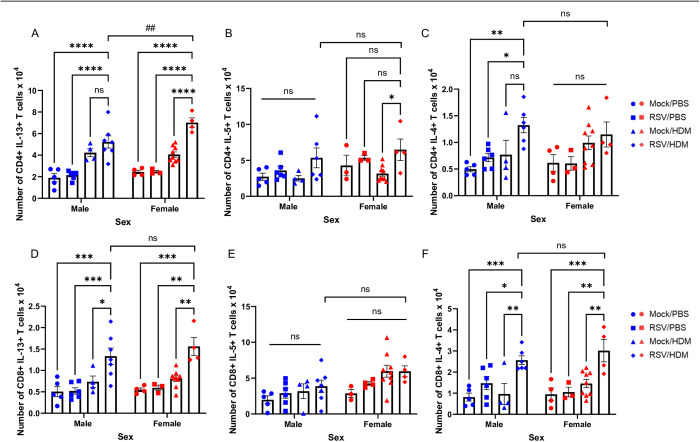
Early-life RSV infection enhances Th2 and Tc2 cell responses to HDM. Mice were treated as in Fig 1A. Absolute count of CD4^+^ T cells **(A-C)** or CD8^+^ T cells **(D-F)** expressing **(A, D)** IL-13, **(B, E)** IL-5, **(C, F)** IL-4. Blue for male, red for female. Data are from a combination of two independent experiments (n = 3–9 per group). Outcomes are presented as mean ± SEM assessed by two-way ANOVA, Tukey’s post hoc test. ns = not significant, *p ≤ 0.05, **or ## p ≤ 0.01, ***p ≤ 0.001, ****p ≤ 0.0001.

Severe lower respiratory tract infection with RSV is associated with increased Th2 polarization in the lung (3–7). Th2-type cytokines, IL-4, IL-5, and IL-13 induce Th2 differentiation; eosinophil differentiation, recruitment and activation; mucus production; and AHR [47–49]. In addition, IL-33 (which was elevated in mice exposed to RSV and HDM), directly stimulates IL-13 production by antigen-experienced CD4^+^ T cells [50]. While CD4^+^ and CD8^+^ T cells expressing IL-13 were increased in RSV → HDM mice compared to RSV → PBS or Mock→HDM mice (Fig 4A and 4D), few changes were noted in populations of IL-5 expressing CD4^+^ or CD8^+^ T cells in males and females, regardless of exposure history (Fig 4B and 4E). Interestingly, compared to CD4^+^ T cells, there were more CD8^+^ T cells expressing IL-4 in RSV → HDM mice (Fig 4C and 4F). On the other hand, there were larger numbers of CD4^+^ T cells expressing IL-13 compared to CD8^+^ T cells expressing IL-13 (Fig 4A and 4D), and these cells are the only cytokine-producing population that was greater in RSV → HDM females compared to males, both in number (Fig 4A) and percent of the parent population producing IL-13 (S6A Fig). Altogether, these data suggest that early-life RSV infection increases HDM-induced expansion of populations of both CD4^+^ and CD8^+^ T cells producing type 2 cytokines, most notably CD4^+^ T cells expressing IL-13, which were greater in RSV → HDM female mice compared to males. Thus, together with ILC2s, these cells are well positioned to enhance eosinophil responses in RSV → HDM female mice.

We examined ST2 levels on both CD4^+^ and CD8^+^ T cells to determine if ST2 expression was associated with the enhanced responses in RSV → HDM mice. While exposure to either RSV → PBS or Mock→HDM was sufficient to increase the number of T cells (particularly CD8^+^ T cells) expressing ST2 at PND24, there was no further increase in either ST2^+^ CD4^+^ or CD8^+^ T cells in RSV → HDM mice (S5F and S5G Fig), suggesting that the enhanced responses in RSV → HDM mice were not associated with selective activation of ST2-expressing T cells. Finally, for both CD4^+^ and CD8^+^ T cells, there was no significant difference in the level of ST2 expression between any treatment group (S5H and S5I Fig). Similarly, no differences in ST2 expression or expansion of cytokine-positive T cells were observed. Together, these data suggest that the enhanced T cell responses in RSV → HDM mice, as well as the greater T cell responses in female mice, were not the result of greater ST2 expression on T cells responding to IL-33.

Oliphant et al. showed that MHCII is expressed by ILC2s, allowing these cells to present antigen and activate CD4^+^ T cells [51]. When we examined MHCII expression on ILC2s in this model, there were more MHCII positive ILC2s in RSV → HDM mice, although on a per cell basis, expression of MHCII did not differ between treatment groups (S5C and S5D Fig). ILC2s were identified based on expression of ST2 (see Fig 2); however, ILC2 levels of ST2 did not differ between treatment groups (S5E Fig). Thus, following RSV → HDM exposure, ILC2s expressing MHCII are increased selectively in female mice, providing evidence that they are well-positioned to activate CD4^+^ T cells in the lung in a sex-specific manner.

### RSV infection-mediated enhanced type 2 allergic lung inflammation is maintained over time

**Fig 5:**
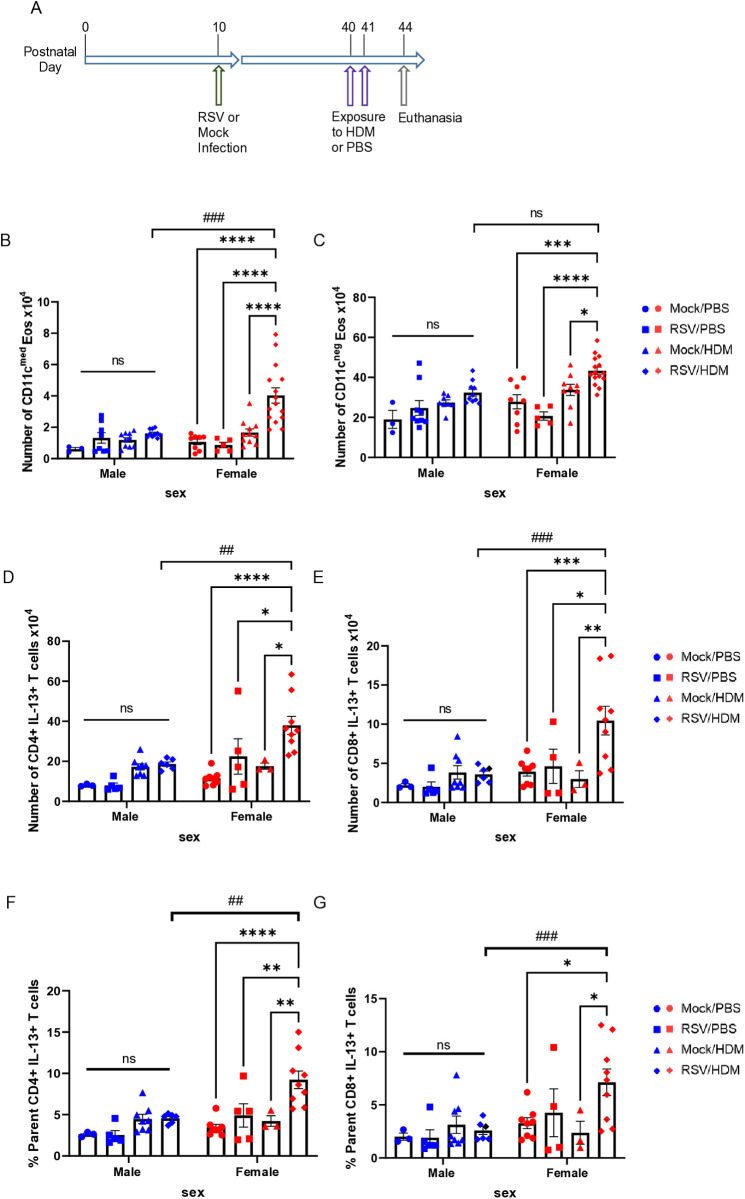
Early-life *RSV infection-mediated enhanced type 2 allergic lung inflammation is maintained over time.* **(A)** Mice were mock infected or exposed to RSV intranasally on PND10 and then treated with PBS or HDM (50ug per dose) intranasally on PND40 and PND41. Seventy-two hours later, lungs were harvested. **(B, C)** Absolute count of CD11c^med^ and CD11c^–^ eosinophils in the lung. Absolute count of IL-13 expressing **(D)** CD4^+^ T cells or **(E)** CD8^+^ T cells. Frequency of **(F)** CD4^+^ T cells or **(G)** CD8^+^ T cells expressing IL-13 within the respective parent populations. Blue for male, red for female. Data are from the combination of two independent experiments (n = 3–9 per group). Outcomes are presented as mean ± SEM assessed by two-way ANOVA, Tukey’s post hoc test. ns = not significant, *p ≤ 0.05, **or ## p ≤ 0.01, ***or ### p ≤ 0.001, ****p ≤ 0.0001.

To examine if the RSV-mediated enhancement of type 2 allergic responses, as well as augmented responses in females, were maintained over time, PND10 mice were infected with RSV and treated with HDM as above except that the interval between the two exposures was expanded from 10 days to one month (Fig 5A). Even after 30 days, the number of CD11c^med^ activated eosinophils was significantly greater in RSV → HDM female mice compared to either treatment alone and to their male counterparts (Fig 5B). The number of eosinophils lacking CD11c in RSV → HDM mice, however, did not differ between males and females (Fig 5C). The number of IL-13^+^ T cells (whether CD4^+^ or CD8^+^) also followed the same trend as in the acute model: female RSV → HDM mice had greater numbers than RSV → PBS or Mock→HDM mice (Fig 5D and 5E) as well as a larger proportion of CD4^+^ or CD8^+^ T cells producing IL-13 (Fig 5F and 5G). Interestingly, sex differences in IL-13-expressng T cells were now more prominent when the time between RSV infection and HDM treatment was extended. Female RSV → HDM mice had significantly more IL-13^+^ T cells compared to males of the same group (Fig 5D and 5E) and enhanced responses in RSV → HDM male mice were largely absent, unlike those in younger mice. No differences were found in T cells expressing IL-4 or IL-5 alone, in contrast to differences in the acute model. These data demonstrate that the ability of RSV to impact allergen-induced inflammation is maintained over time, reflected in enhanced responses of eosinophils as well as T cells with the potential to produce IL-13.

### Increased HDM-induced lung inflammation and KLRG1– ILC2s in female mice infected in early life with RSV

**Fig 6:**
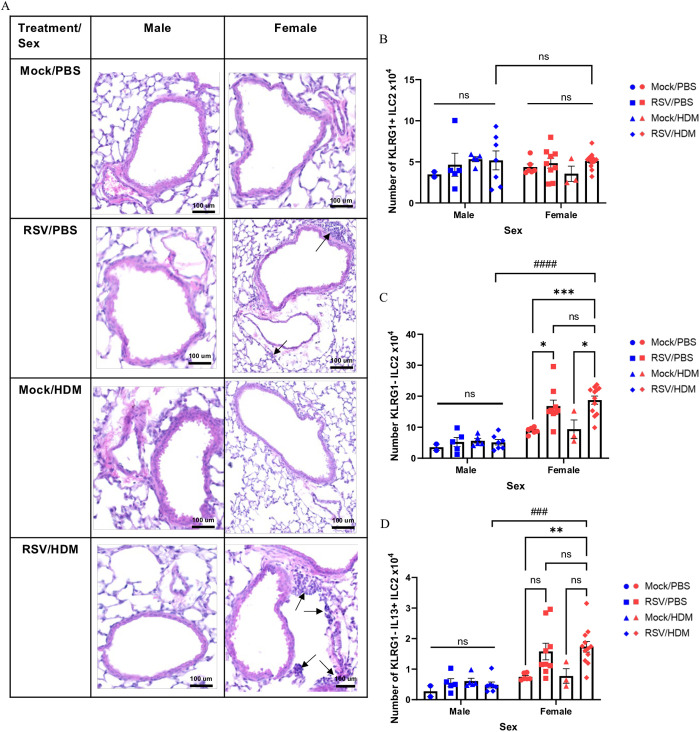
*Increased HDM-induced lung inflammation and KLRG1*^–^ *ILC2s in female mice infected in early life with RSV.* Mice were treated as in Fig 5A and lungs harvested for **(A)** histology or **(B-D)** flow cytometry 72h after HDM exposure. **(A)** H&E staining showing representative sections from male and female mice in each group. Images are representative of 16 mice from two independent experiments (n = 2-4 per group). Arrows mark the presence of cell infiltration around the airways. Magnification x10. Scale bar = 100 um. Absolute count of **(B)** KLRG1^+^ ILC2s, **(C)** KLRG1^–^ ILC2s, and **(D)** KLRG1^–^ ILC2s expressing IL-13 in males and females. Blue for male, red for female. Data in B-D are from the combination of two independent experiments (n = 3–12 per group). Outcomes are presented as the mean ± SEM assessed by two-way ANOVA, Tukey’s post hoc test. ns = not significant, *p ≤ 0.05, **p ≤ 0.01, ***or ###p ≤ 0.001, ####p ≤ 0.0001.

Lung inflammation and cell infiltration into the lung were also assessed by H&E staining. While modest in all mice, inflammation was most prominent in female RSV → HDM mice, in which cell infiltration was present around both blood vessels and airways (Figs 6A and S7D), whereas inflammation in their male counterparts did not differ from untreated controls. Interestingly, RSV → PBS female mice also showed a trend for increased inflammation (Fig 6A). Unexpectedly, KLRG1^–^ (but not KLRG1^+^) ILC2s were increased in females, but not males, infected one-month prior with RSV, whether exposed to HDM or not (Fig 6B and 6C). Similar increases in IL-13-expressing KLRG1^–^ ILC2s were also apparent in RSV → PBS female mice, though the increase did not reach statistical significance (Fig 6D). Interestingly, IL-33 levels in the lung, which had returned to baseline 10 days after RSV infection, increased for a second time, selectively in adult female mice (Fig 3B).

Given the rapid changes that occur in the murine lung in the first weeks of life [38], we also examined whether exposing mice to RSV at an even younger age (PND4), would impact the inflammatory response in adults. In fact, the number of KLRG1^–^ ILC2s was greater when neonates were infected with RSV at PND4, compared to PND10, while the number of KLRG1^+^ ILC2s was not affected by age of RSV infection (S7A and S7B Fig). Whether the adult mice were exposed to HDM or not had no impact on the number of KLRG1^–^ ILC2s. While CD11c^med^ activated eosinophils were increased only in RSV-HDM mice, these were not further elevated in mice exposed to RSV at PND4 (S4C Fig), providing evidence that the increased number of KLRG1^–^ ILC2s was not sufficient to drive HDM-induced upregulation of CD11c and activation of eosinophils [34]. These data suggest that early-life RSV infection modifies the lung microenvironment such that KLRG1^–^ ILC2s increase several weeks later selectively in adult female mice, in a manner that is sensitive to age of infection - with earlier infection, driving even greater expansion.

### AHR is present only in female mice with RSV infection prior to HDM exposure

**Fig 7:**
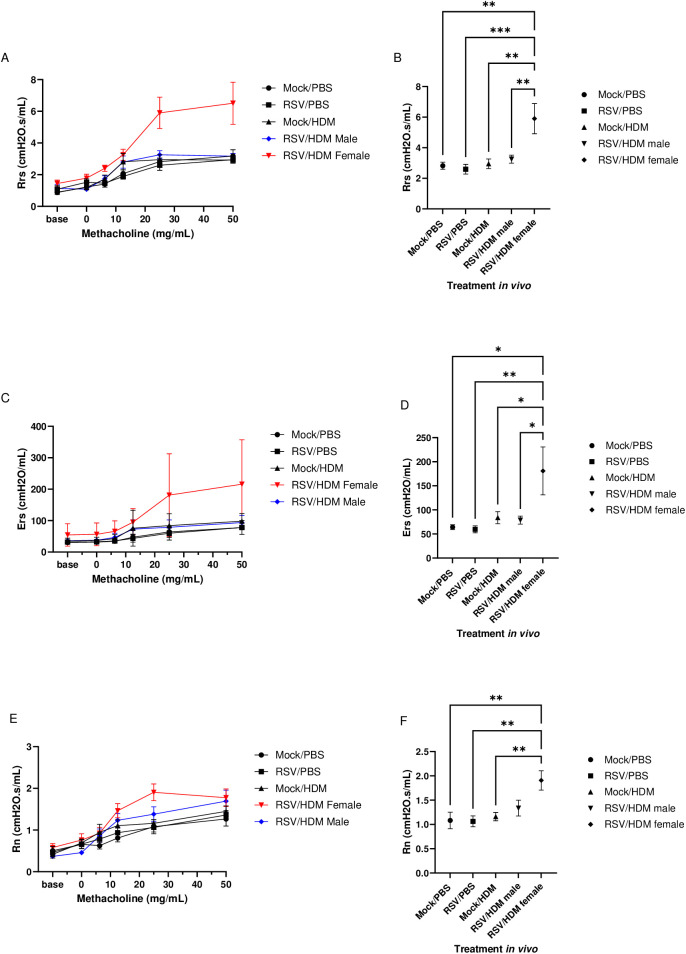
Early-life RSV infection is required for AHR induction following acute HDM exposure only in female mice. Mice were treated as in Fig 5A and lung function assessed 72h after HDM exposure. Respiratory system resistance (Rrs): **(A)** methacholine dose response and **(B)** 25 mg/mL dose. Respiratory elastance (Ers): **(C)** methacholine dose response and **(D)** 25mg/mL dose. Newtonian resistance (Rn): **(E)** methacholine dose response and **(F)** 25mg/mL dose. Data are from the combination of two independent experiments (n = 6–8 per group). In mock/PBS, RSV/PBS and mock/HDM no sex differences were observed; therefore, male and female mice were combined in these groups. Outcomes are presented as the mean ± SEM assessed by one-way ANOVA, Tukey’s post hoc test. *p ≤ 0.1, **p ≤ 0.01, ***= p ≤ 0.001.

We examined effects of early-life RSV infection on lung function induced upon exposure to HDM in adult mice. As HDM was used acutely in this model, Mock→HDM mice were not expected to show altered lung function and this was not observed in any parameters measured (Fig 7). Both respiratory system resistance (Rrs) and respiratory elastance (Ers) (measures of total lung resistance and lung stiffness, respectively) were significantly greater in RSV → HDM female mice, compared to Mock→PBS, RSV → PBS or Mock→HDM mice (Fig 7A–7D). Rrs and Ers were also significantly greater in RSV → HDM females compared to their male counterparts (Fig 7A–7D), in which no changes compared to control (Mock→PBS, RSV → PBS or Mock→HDM) mice were apparent. Similarly, Newtonian resistance (Rn) (a measure of resistance in the larger conducting airways) was significantly greater in RSV → HDM female mice, compared to Mock→PBS, RSV → PBS or Mock→HDM mice (Fig 7E and 7F). Rn was not significantly greater in RSV → HDM females compared to their male counterparts (Fig 7E and 7F), although a trend for RSV → HDM female mice to be higher was present. These results show that lung function, similar to that in murine models of asthma [52], is impacted long term by early-life RSV infection, particularly in female mice. Altogether, these data provide evidence that RSV modifies the lung environment, enhancing inflammatory responses and inducing AHR upon acute allergen exposure in females, in a manner that persists for several weeks.

## Discussion

A strong association between RSV bronchiolitis and subsequent wheezing and asthma has been recognized for decades [53]. However, new data link even mild disease in early life to increased risk of asthma at 5 years of age, suggesting that the link between early-life RSV exposure and asthma is more pervasive [54,55]. There is great interest in limiting early-life RSV infection, to improve respiratory health of infants in the short term, and to reduce long-term outcomes of respiratory morbidity (i.e., wheezing, asthma) [56–58]. Thus, understanding the link between these diseases may help guide the development of effective treatments. In the current study, we demonstrate that infection of young mice (PND10) with RSV followed by an acute 2-day intranasal exposure to the common allergen, HDM, promoted perivascular and peribronchial inflammation as well as eosinophil influx and activation, ILC2 expansion, and AHR. Inflammatory responses were greater in female mice compared to those in males; in particular, the subset of ILC2s lacking expression of KLRG1 and producing type 2 cytokines expanded more in female mice, in agreement with data from others showing that androgen signaling negatively regulates ILC2s and that adult female mice have greater levels of KLRG1^–^ ILC2s [23,25,42]. We have not, however, ruled out a role for androgen signaling in the regulation of KLRG1^+^ ILC2s in this model, since IL-5-producing KLRG1^+^ ILC2s were also significantly lower in RSV → HDM male mice compared to their female counterparts.

In young mice previously infected with RSV and exposed to HDM 10 days later, expansion of ILC2s was enhanced in female mice. However, when ILC2s were quantified in adult mice, larger numbers of KLRG1^–^ ILC2s were present in all female mice infected with RSV as neonates, whether they were subsequently exposed to HDM or not. Since this sex-specific expansion of ILC2s was observed in both the shorter (PND10 to PND24) and more prolonged models (PND10 to PND44), it is unlikely these effects can be attributed *entirely* to hormones, since gonadal hormone production is very limited in young mice [59]. Data from our more prolonged model provide insight into which cells may contribute to production of several type 2 cytokines in the lung during RSV-dependent enhancement of acute HDM-induced type 2 inflammatory response. In this model, the sex differences in T cells were more apparent when the time between RSV infection and allergen challenge was extended. As speculated for the ILC2s, heightened sex differences in the more prolonged model could be attributed to the age of the mice and the effect of hormones as the mice sexually mature.

Another possibility we examined was whether RSV infection increased levels of IL-33 in the lungs over time as the mice mature, which would be predicted to increase ILC2 levels, even in the absence of HDM exposure. Werder et al showed that BALF levels of IL-33 were significantly increased 72h after repeated exposure to CRE, specifically in mice previously infected with pneumonia virus of mice (PVM) as neonates [60]. Even in the absence of further PVM or CRE exposure, BALF IL-33 levels continued to increase for at least 28 days. Werder et al did not examine the impact of elevated IL-33 on ILC2s and the sex of mice was not presented in this study [60]. However, our data show that the increase in KLRG1^–^ ILC2s in adult female mice aligned with increased levels of IL-33, selectively in mice previously infected with RSV as neonates and suggest that early-life RSV infection may influence the impact of sex hormones on IL-33 levels and/or KLRG1^–^ ILC2s in the lung - further supported by the fact that KLRG1^–^ ILC2s were not similarly elevated in younger RSV → PBS PND24 mice, prior to full sexual maturity. Nevertheless, while KLRG1^–^ ILC2s were elevated in adult female mice, even in the absence of HDM, they were not sufficient to induce a change in AHR, which was increased in female mice, but only after HDM exposure. In fact, AHR in these mice was more closely aligned with elevated numbers of CD4^+^ and CD8^+^ T cells expressing IL-13. Further work is required to define mechanisms by which AHR develops selectively in female mice following acute HDM exposure and to better understand why ILC2s increase in female mice over time following early-life RSV infection.

Elevated levels of CD8^+^ T cells expressing IL-4 in nasal aspirates are associated with increased disease severity in RSV-infected infants [61]. In our murine model, CD8^+^IL-4^+^ T cells triggered by RSV infection may respond to IL-33 released by epithelial cells following acute HDM exposure [62], in an antigen-independent manner through ST2 signaling [63]. The increase in IL-13^+^ CD4^+^ T cells we observed agrees with the findings from Guo et al. and Endo et al. who showed, respectively, that effector Th2 cells and effector-memory Th2 cells promote type 2 inflammation by producing IL-13 and/or IL-5 in a TCR-*independent* manner in response to IL-33 [50,64]. However, although IL-33 levels in the lung were significantly increased within hours of HDM delivery in RSV treated young mice, the increase in IL-33 did not appear to directly impact T cell expansion as ST2^+^ T cell numbers, as well as the quantity of ST2 expressed per cell, were not greater in RSV → HDM mice, nor was their number greater in females compared to males.

The role for other innate cytokines in enhanced responses to HDM in RSV-exposed mice has not, however, been excluded. IL-25 has been shown to play an important role in regulating the inflammatory response to RSV [65,66]. For example, data from Peterson et al. showed that IL-25 and its receptor IL-17RB are expressed in the lungs of RSV-infected BALB/c mice and that knocking out IL-17RB reduces pathology in these mice [65]. Moreover, Lee et al. showed that the innate cytokine, TSLP, is elevated in the lungs of RSV-infected mice and that TSLP receptor (TSLPR) knock-out mice have reduced levels of IL-13 as well reduced airway resistance [67]. In skin inflammation models, CD4^+^ T cell specific expression of TSLPR is critical for infiltration of both CD4^+^ T cells as well as eosinophils [68]. Thus, in our model, the expansion of T cells could be in part due to the release of other innate cytokines upon RSV infection leading to larger numbers of T cells in the lung. Another possibility is that the effects of IL-33 on T cells may be indirect. For example, enhanced production of IL-13 by ILC2s, particularly in ILC2s lacking expression of KLRG1, in female mice exposed to RSV → HDM could promote dendritic cell activity [69,70], enhancing expansion and cytokine production of both CD4^+^ and CD8^+^ T cells. In this scenario, increased T cell responses would correspond primarily to RSV-specific effector/memory T cells in combination with a smaller contribution from newly differentiating HDM-specific T cells.

Our cumulative results demonstrate that, compared to males, female mice are impacted to a greater extend by early-life RSV infection prior to acute HDM exposure. Our data show greater numbers of eosinophils, ILC2s, and T cells, as well as increased lung infiltration and AHR in females compared to males. Malinczak et al. showed that RSV infection prior to CRE sensitization and challenge leads to increased airway hyperactivity, mucus production, and IL-13 mRNA levels in male mice compared to females [11]. These differences could be attributed to the strain of RSV used: RSV A2 with Line 19 fusion protein in their work vs. RSV A2 in ours. RSV A2 with Line 19 fusion protein leads to greater viral load, pulmonary IL-13 and mucin, and lung resistance compared to the A2 strain [71]. Another important difference is that mice used in the Malinczak et al study were sensitized and challenged with CRE, to induce CRE-specific Th2 adaptive immunity and allergic airways disease, and all of our outcomes were within days of an acute exposure to HDM, a time point in which HDM-specific Th2 adaptive immunity was not fully established [11]. Understanding how RSV strain differences and sex interact, both in neonates and adults, will be an important consideration to better understand effects of lower respiratory tract RSV infection in both children and adults.

## Conclusions

Altogether, data from this RSV → HDM model suggest that the increased number of KLRG1^–^ IL-13^+^ ILC2s as well as IL-13^+^ T cells, may be responsible for the increased numbers of activated CD11c^med^ eosinophils in lungs of RSV-infected neonates subsequently exposed to acute HDM delivery. This may be through interactions with innate cytokines such as IL-33 though our data do not rule out a role for other innate cytokines, such as IL-25 and TSLP. Larger numbers of IL-5^+^ ILC2s in female mice may help explain why recruitment of CD11c^–^ eosinophils was greater in these mice. Moreover, there were larger numbers of T cells producing type 2 cytokines in response to acute HDM exposure in mice infected 10 days prior with RSV. We speculate that these T cells respond to a type 2 skewed lung environment “primed” by RSV infection [72], driving them to respond more strongly to a second encounter with a similar innate cytokine milieu induced by HDM exposure. The precise antigen specificity of these T cells is not yet known. This body of work also sheds light on important sex differences that occur in type 2 inflammatory responses associated with early-life RSV infection and furthers our understanding of how “training” of cells of the innate immune system by virus infection may have enduring local effects on immune responses. As the prevalence and severity of asthma has been on the rise for many years and the impact of new treatment options that prevent severe RSV disease on long-term lung morbidity is not known, a better understanding of the connection between early-life events and subsequent allergen exposures can contribute to the development of better prophylactic and/or treatment strategies to mitigate these risks that may differ between the sexes.

## Supporting information

S1 File Tables A-C. Antibodies used for (A) eosinophil and macrophages (B) ILC2; and (C) T cell multi-color flow cytometry.
(TIF)

S1 Fig Interstitial macrophages, but not neutrophils or alveolar macrophages, are increased by acute HDM delivery in mice infected with RSV in early life. Mice were treated as in Fig 1A. **(A)** Flow cytometry gating strategy to identify neutrophils, alveolar macrophages and interstitial macrophages. Absolute count of **(B)** neutrophils, **(C)** alveolar macrophages (AM), and **(D)** interstitial macrophages (IM). Blue for male, red for female. Data are from the combination of two (C, D) or three (B) independent experiments (n = 4–16 per group). Outcomes are presented as mean ± SEM assessed by two-way ANOVA, Tukey’s post hoc test. ns = not significant, ***p ≤ 0.001, ****p ≤ 0.0001.
(TIF)

S2 Fig Flow gating strategy to identify type 2 immune cells. **(A)** ILC2 were stained using AF488-IL-13, APC-IL-5, EF-450-Thy1.2, PECy7-CD127, PerCP-eF710-ST2, KLRG1-BV605, CD45.2-BUV395, and a combination of PE-conjugated antibodies to CD3e, CD11c, CD11b, CD49b, CD45R, TCRyD, Ly6G, and FCeRa and gated as depicted. Inset in last panel shows FMO controls for IL-5 and IL-13. **(B)** T cells were stained using FITC-CD4, PerCP-Cy5.5-CD8, V500-CD3, BUV395-CD45.2 and gated as depicted. Insets in last 2 panels show FMO controls for IL-5, IL-13, IL-4.
(TIF)

S3 Fig Kinetics of RSV detection in neonates infected at PND10. Mice were infected on PND10 and RSV quantified by qRT-PCR as described in the methods. Data are from the combination of 11 experiments (n = 6–17 mice per group).
(TIFF)

S4 Fig Early-life RSV infection enhances HDM-induced ILC2 responses, selectively in females. Mice were treated as in Fig 1A. Frequency of KLRG1^+^ ILC2s producing IL-5 (A) or IL-13 (C) or KLRG1^–^ ILC2s producing IL-5 (B) or IL-13 (D) within the respective parent population. Blue for male, red for female. Data are from the combination of two independent experiments (n = 3–8 per group). Outcomes are presented as mean ± SEM assessed by two-way ANOVA, Tukey’s post hoc test. ns = not significant, *or #p ≤ 0.05, **p ≤ 0.01, ***p ≤ 0.001, ****p ≤ 0.0001.
(TIFF)

S5 Fig Lung cell cytokine production and characterization of ILC2 and T cell phenotype. Mice were treated as in Fig 1A. Lungs were harvested and **(A, B)** cultured ex vivo or **(C-I)** assessed by flow cytometry. Quantity of **(A)** IL-13 or **(B)** IL-5 from saline or IL-33 cultured lung cells. **(C)** Absolute count of KLRG1^–^ ILC2s expressing MHCII. Median fluorescence intensity (MFI) of **(D)** MHCII or **(E)** ST2 on ILC2s. Absolute count of **(F)** CD4^+^or **(G)** CD8^+^ T cells expressing ST2. MFI of ST2 on **(H)** CD4^+^or **(I)** CD8^+^ T cells. Blue for male, red for female. Data are from the combination of two independent experiments (n = 4–12 per group). Outcomes are presented as mean ± SEM assessed by two-way ANOVA, Tukey’s post hoc test. ns = not significant, *p ≤ 0.05, **p ≤ 0.01, ***p ≤ 0.001, ****p ≤ 0.0001.
(TIFF)

S6 Fig Early-life RSV infection enhances Th2 and Tc2 response to HDM, particularly in females. Mice were treated as in Fig 1A. Frequency of CD4^+^ T cells (A-C) or CD8 + T cells ((D-F) expressing **(A, D)** IL-13, **(B, E)** IL-5, or **(C, F)** IL-4 within the respective parent population. Blue for male, red for female. Data are from the combination of two independent experiments (n = 3–9 per group). Outcomes are presented as mean ± SEM assessed by two-way ANOVA, Tukey’s post hoc test. ns = not significant, *p ≤ 0.05, **or ##p ≤ 0.01.
(TIFF)

S7 Fig *KLRG1*^–^ *ILC2s are greater in mice infected with RSV at PND4 compared to PND10.* Mice were infected with RSV on PND4 or PND10 and then treated with PBS or HDM on PND40 and PND41. Seventy-two hours later, lungs were harvested. Absolute count of **(A)** KLRG1^+^ and **(B)** KLRG1^–^ ILC2s. **(C)** Absolute count of CD11c^med^ activated eosinophils. n = 5–7 per group. **(D)** Histological differences were quantified by two blinded individuals using a scale from 1 (very little cell infiltration) to 3 (higher amount of cellular infiltration), n = 2–4 per group, error bars for Mock/PBS and Mock/HDM represent range. Outcomes are presented as mean ± SEM assessed by two-way ANOVA, Tukey’s post hoc test. *or # p ≤ 0.05, **** p ≤ 0.0001.
(TIFF)

S1 Data Data source file for Figs. Experimental data for Figs 1–7, as well as S1 and S3-S7 Figs.
(XLSX)
