# Supplementary figures and images for: Early-life RSV infection modulates innate immune events, preferentially enhancing allergen-induced type 2 lung inflammation in females
Source: PLoS Pathog. 2025 Jul 21;21(7):e1013340. doi: 10.1371/journal.ppat.1013340 (PMC12310010; doi:10.1371/journal.ppat.1013340)

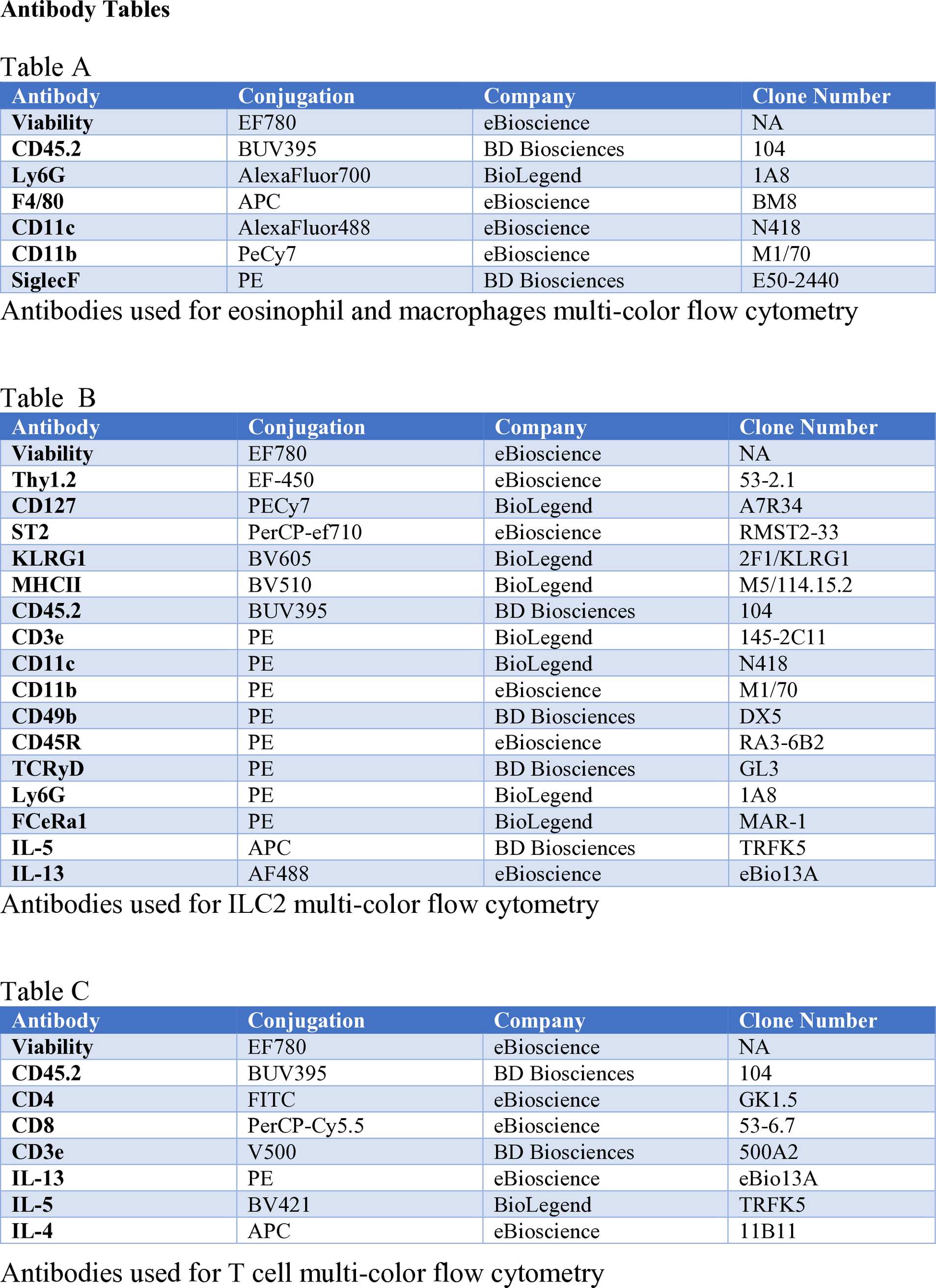

Supplement: S1 File — Antibodies used for (A) eosinophil and macrophages (B) ILC2; and (C) T cell multi-color flow cytometry. (TIF) [file ppat.1013340.s001.tif]

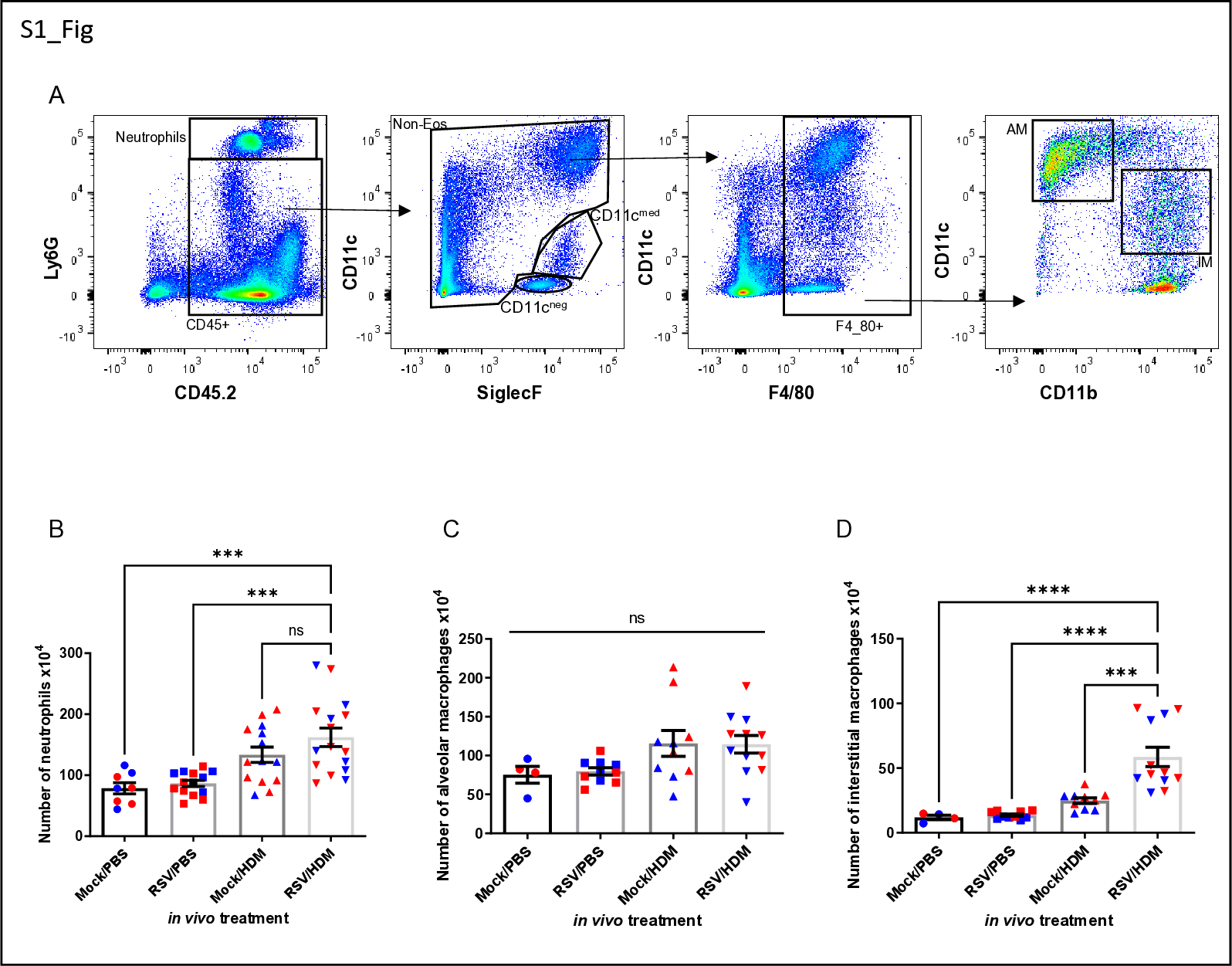

Supplement: S1 Fig — Mice were treated as in Fig 1A. (A) Flow cytometry gating strategy to identify neutrophils, alveolar macrophages and interstitial macrophages. Absolute count of (B) neutrophils, (C) alveolar macrophages (AM), and (D) interstitial macrophages (IM). Blue for male, red for female. Data are from the combination of two (C, D) or three (B) independent experiments (n = 4–16 per group). Outcomes are presented as mean ± SEM assessed by two-way ANOVA, Tukey’s post hoc test. ns = not significant, ***p ≤ 0.001, ****p ≤ 0.0001. (TIF) [file ppat.1013340.s002.tif]

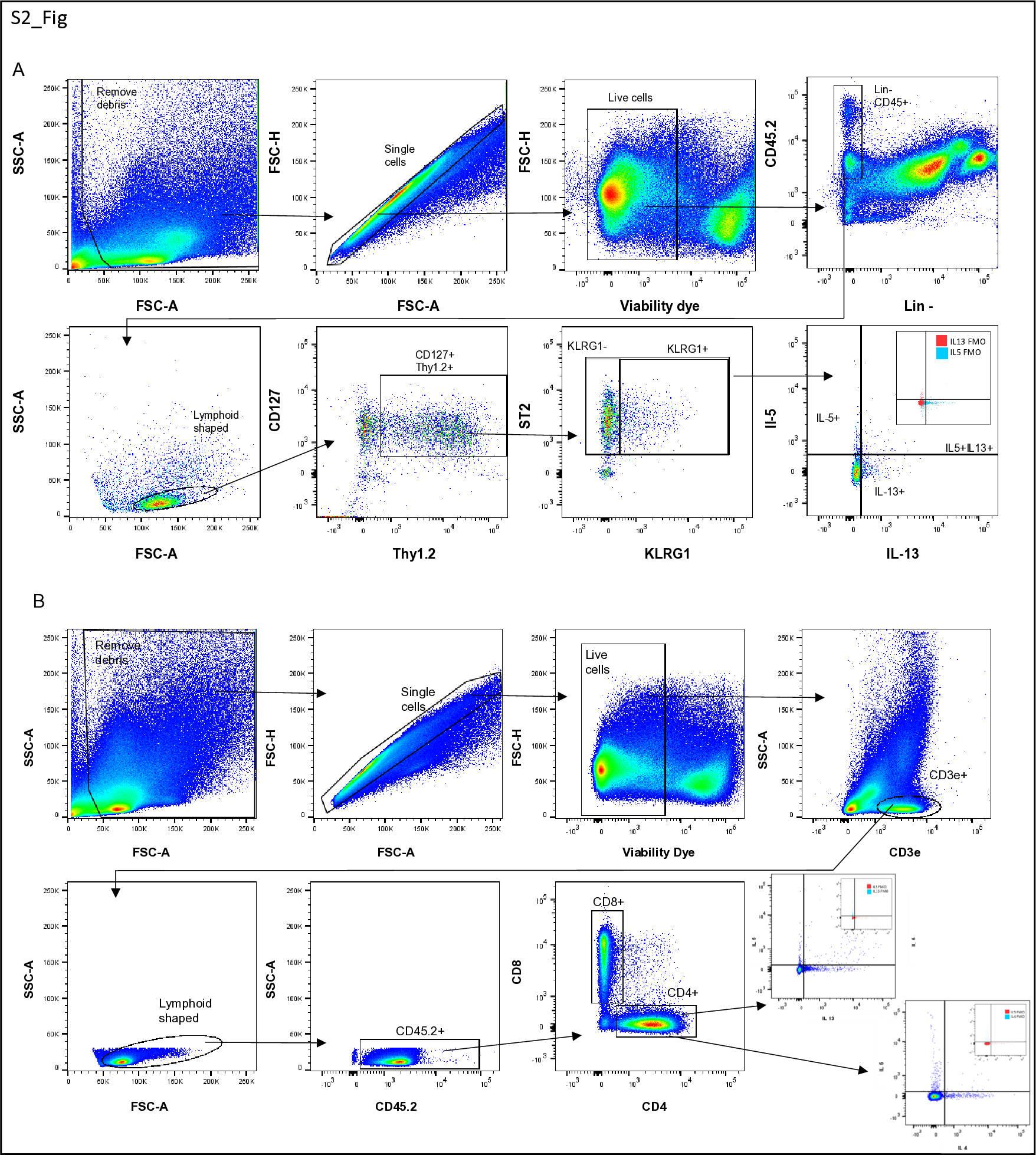

Supplement: S2 Fig — (A) ILC2 were stained using AF488-IL-13, APC-IL-5, EF-450-Thy1.2, PECy7-CD127, PerCP-eF710-ST2, KLRG1-BV605, CD45.2-BUV395, and a combination of PE-conjugated antibodies to CD3e, CD11c, CD11b, CD49b, CD45R, TCRyD, Ly6G, and FCeRa and gated as depicted. Inset in last panel shows FMO controls for IL-5 and IL-13. (B) T cells were stained using FITC-CD4, PerCP-Cy5.5-CD8, V500-CD3, BUV395-CD45.2 and gated as depicted. Insets in last 2 panels show FMO controls for IL-5, IL-13, IL-4. (TIF) [file ppat.1013340.s003.tif]

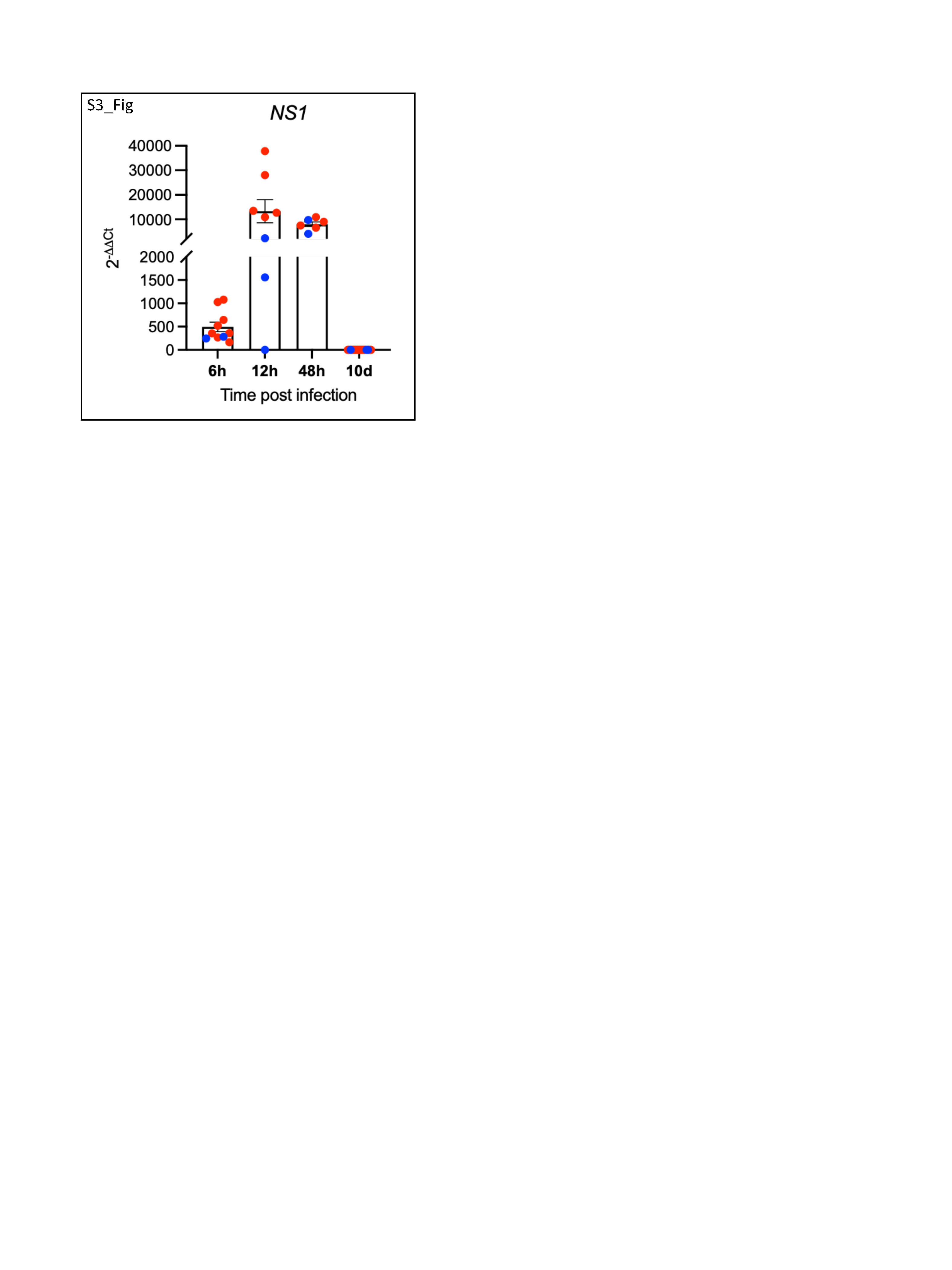

Supplement: S3 Fig — Mice were infected on PND10 and RSV quantified by qRT-PCR as described in the methods. Data are from the combination of 11 experiments (n = 6–17 mice per group). (TIFF) [file ppat.1013340.s004.tiff]

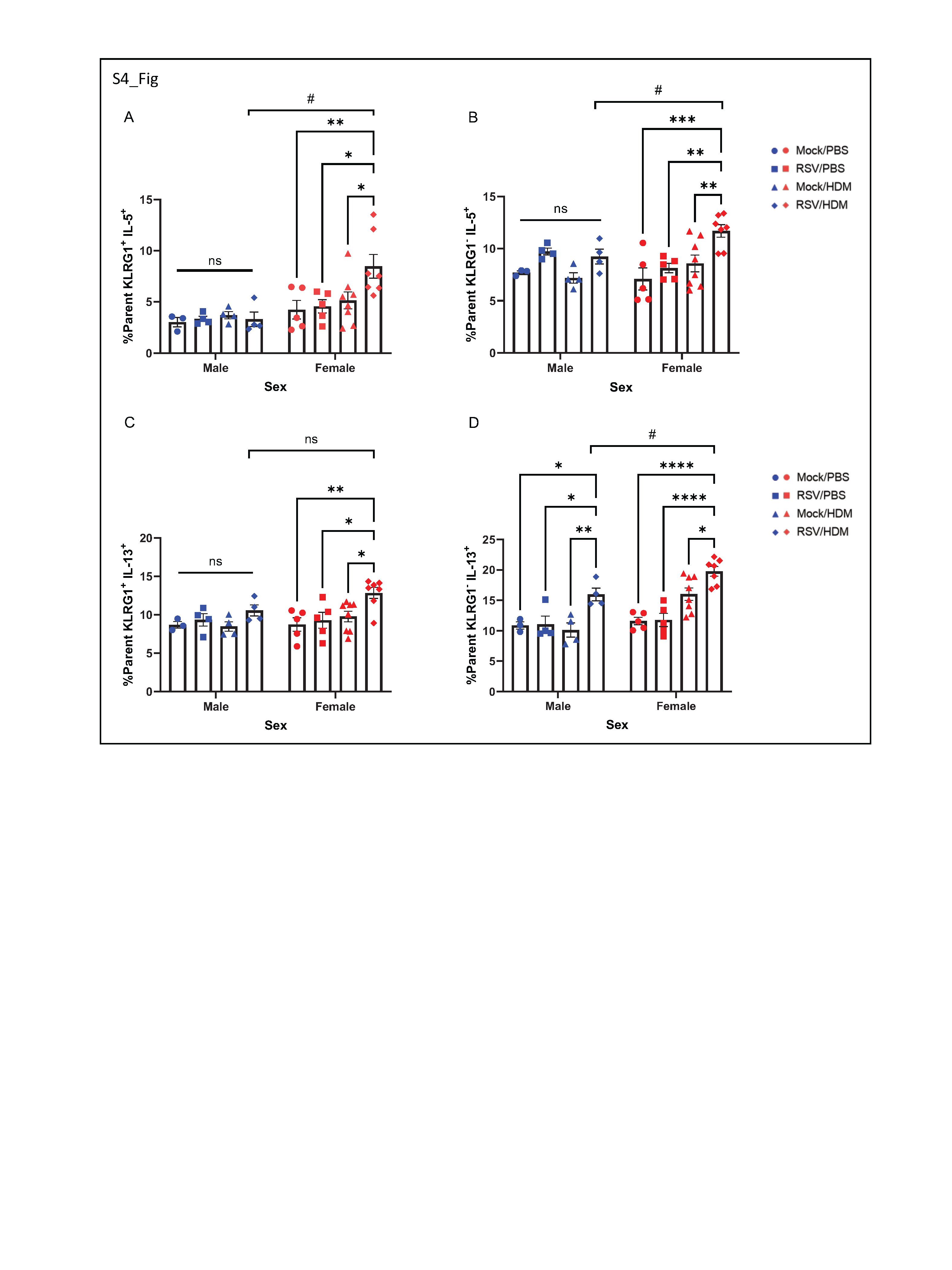

Supplement: S4 Fig — Mice were treated as in Fig 1A. Frequency of KLRG1+ ILC2s producing IL-5 (A) or IL-13 (C) or KLRG1– ILC2s producing IL-5 (B) or IL-13 (D) within the respective parent population. Blue for male, red for female. Data are from the combination of two independent experiments (n = 3–8 per group). Outcomes are presented as mean ± SEM assessed by two-way ANOVA, Tukey’s post hoc test. ns = not significant, *or #p ≤ 0.05, **p ≤ 0.01, ***p ≤ 0.001, ****p ≤ 0.0001. (TIFF) [file ppat.1013340.s005.tiff]

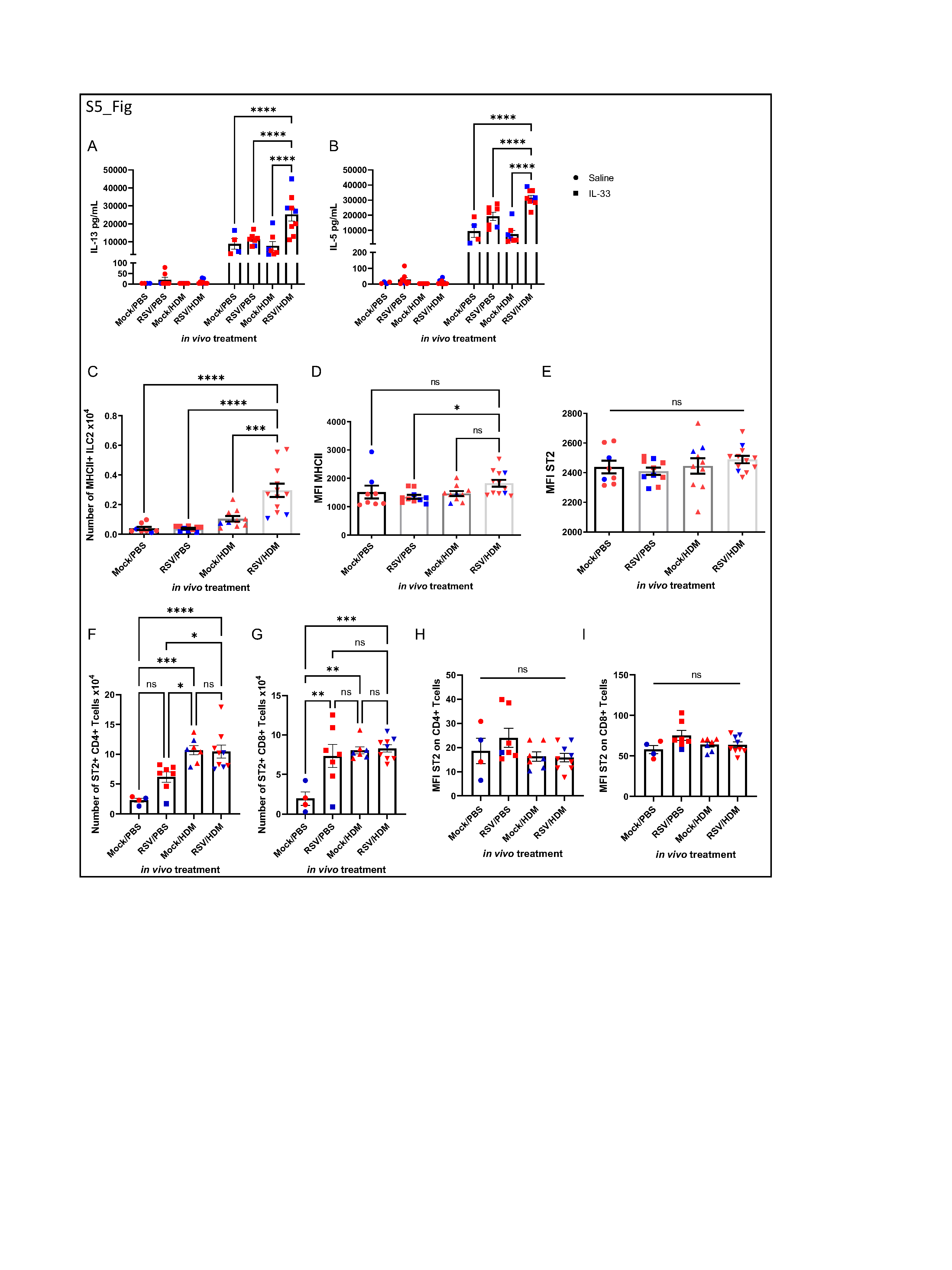

Supplement: S5 Fig — Mice were treated as in Fig 1A. Lungs were harvested and (A, B) cultured ex vivo or (C-I) assessed by flow cytometry. Quantity of (A) IL-13 or (B) IL-5 from saline or IL-33 cultured lung cells. (C) Absolute count of KLRG1– ILC2s expressing MHCII. Median fluorescence intensity (MFI) of (D) MHCII or (E) ST2 on ILC2s. Absolute count of (F) CD4+or (G) CD8+ T cells expressing ST2. MFI of ST2 on (H) CD4+or (I) CD8+ T cells. Blue for male, red for female. Data are from the combination of two independent experiments (n = 4–12 per group). Outcomes are presented as mean ± SEM assessed by two-way ANOVA, Tukey’s post hoc test. ns = not significant, *p ≤ 0.05, **p ≤ 0.01, ***p ≤ 0.001, ****p ≤ 0.0001. (TIFF) [file ppat.1013340.s006.tiff]

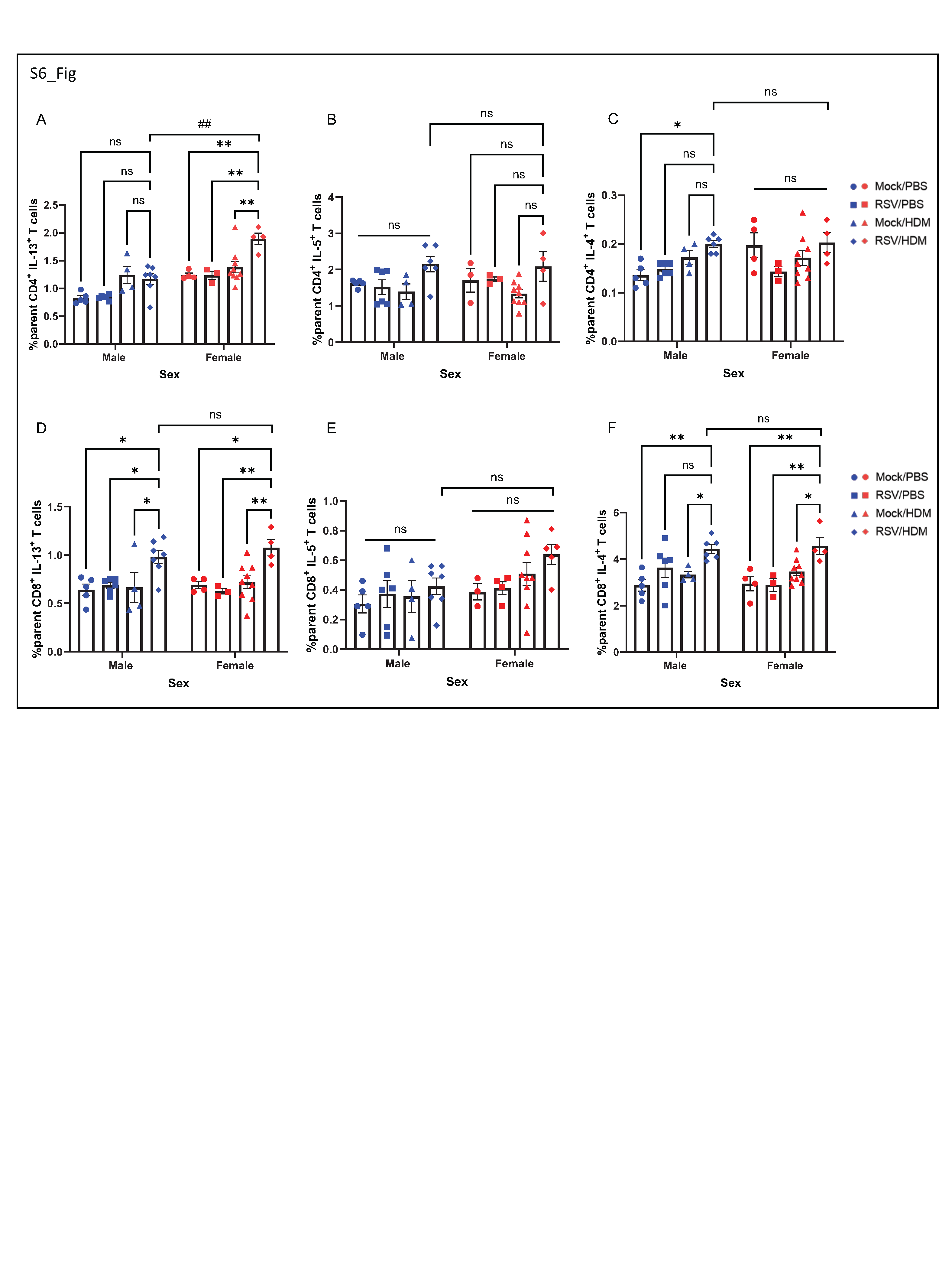

Supplement: S6 Fig — Mice were treated as in Fig 1A. Frequency of CD4+ T cells (A-C) or CD8 + T cells ((D-F) expressing (A, D) IL-13, (B, E) IL-5, or (C, F) IL-4 within the respective parent population. Blue for male, red for female. Data are from the combination of two independent experiments (n = 3–9 per group). Outcomes are presented as mean ± SEM assessed by two-way ANOVA, Tukey’s post hoc test. ns = not significant, *p ≤ 0.05, **or ##p ≤ 0.01. (TIFF) [file ppat.1013340.s007.tiff]

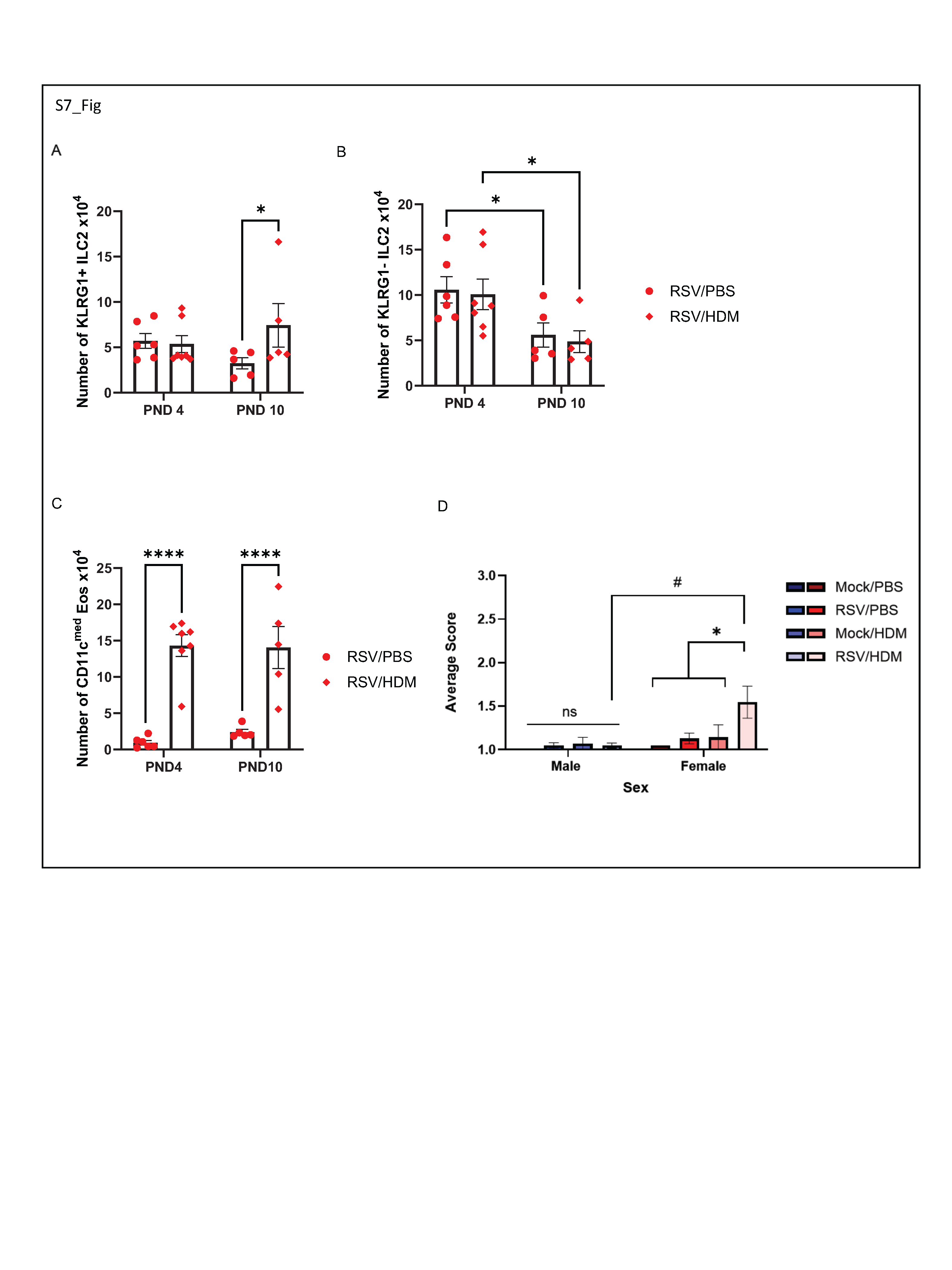

Supplement: S7 Fig — Mice were infected with RSV on PND4 or PND10 and then treated with PBS or HDM on PND40 and PND41. Seventy-two hours later, lungs were harvested. Absolute count of (A) KLRG1+ and (B) KLRG1– ILC2s. (C) Absolute count of CD11cmed activated eosinophils. n = 5–7 per group. (D) Histological differences were quantified by two blinded individuals using a scale from 1 (very little cell infiltration) to 3 (higher amount of cellular infiltration), n = 2–4 per group, error bars for Mock/PBS and Mock/HDM represent range. Outcomes are presented as mean ± SEM assessed by two-way ANOVA, Tukey’s post hoc test. *or # p ≤ 0.05, **** p ≤ 0.0001. (TIFF) [file ppat.1013340.s008.tiff]
